# Advances in Processing Techniques and Determinants of Sweet Potato Starch Gelatinization

**DOI:** 10.3390/foods14040545

**Published:** 2025-02-07

**Authors:** Songtao Yang, Wentao Hu, Shuai Qiao, Wei Song, Wenfang Tan

**Affiliations:** 1 Sichuan Germplasm Resources Center, Crop Research Institute, Sichuan Academy of Agricultural Sciences, Chengdu 610066, China; yost@scsaas.cn (S.Y.); qiaoshuai@scsaas.cn (S.Q.); songweizws@163.com (W.S.); 2 Environmentally Friendly Crop Germplasm Innovation and Genetic Improvement Key Laboratory of Sichuan Province, Chengdu 610066, China; 3 College of Life Science, Nanchang University, Nanchang 330031, China; huwentao0306@163.com

**Keywords:** sweet potato starch, gelatinization, processing techniques, food applications

## Abstract

Sweet potato starch is an important source of starch in food processing, but its natural functionality is relatively limited, restricting its performance in certain applications. Gelatinized sweet potato starch, with enhanced structural and functional properties, has broader potential applications in food products. During the gelatinization process, the crystalline structure of sweet potato starch changes, making it suitable for use in various food formulations. Gelatinized sweet potato starch can be produced through techniques such as moist heat processing, extrusion, and spray drying, with the gelatinization effect influenced by factors such as moisture content and temperature. This review summarizes the gelatinization techniques and influencing factors for sweet potato starch, highlighting how structural changes under different conditions affect the quality of the final food products. Understanding these techniques and influencing factors helps optimize the gelatinization process of sweet potato starch, enhancing its application in foods such as noodles and baked goods. This knowledge provides theoretical support and practical guidance for the further utilization of sweet potato starch in the food industry.

## 1. Introduction

Sweet potatoes (*Ipomoea batatas*) are a versatile tuber crop highly valued for their nutritional content, which includes carbohydrates, fiber, vitamins, and minerals [1,2]. These nutrients make sweet potatoes a vital food source for many populations. One of the key components of sweet potatoes is their starch, accounting for approximately 40.1–55.1% of their dry weight [3]. However, the natural structure of sweet potato starch, characterized by its highly ordered crystalline organization, restricts its ability to swell and absorb water [4].

To enhance the functional properties of natural sweet potato starch, physical or chemical modifications are often applied, with gelatinization being a commonly utilized method [5,6]. During this process, sweet potato starch undergoes structural changes under the influence of heat and water [7]. This exposure disrupts the crystalline areas, facilitating increased water absorption and granule swelling [7]. The resultant increase in starch viscosity and gel-forming capacity leads to a structure better suited to various processing conditions and applications [8]. Thus, gelatinized sweet potato starch proves essential for improving texture, stability, and hydration characteristics in food products.

Various processing techniques, including moist heat treatment, extrusion, microwave heating, and high-pressure processing, can induce the gelatinization of sweet potato starch [9,10,11,12,13]. Each method affects the extent of gelatinization differently, influenced by factors such as humidity, temperature, and processing duration. For example, boiling maximizes water absorption, promoting complete starch gelatinization, while steaming results in a lower degree of gelatinization due to reduced direct contact with water [14,15]. Additionally, high-pressure processing provides a non-thermal approach to starch gelatinization, preserving more nutrients and bioactive compounds [16]. The diversity in these processing techniques significantly impacts the starch’s structural transformation and ultimately affects the quality and functionality of the final sweet potato starch product. However, the current understanding of gelatinization processing methods and influencing factors is still limited.

This review aims to provide a comprehensive overview of the processing techniques used to induce gelatinization in sweet potato starch, along with the influencing factors and food applications. It highlights several discussions on the importance of optimizing the use of gelatinized sweet potato starch or addressing related issues, thereby supporting the development of high-quality food items utilizing this starch.

## 2. Processing Techniques for Gelatinization of Sweet Potato Starch

The gelatinization of sweet potato starch is primarily achieved through various moist thermal processing methods, including boiling, baking, microwave heating, and frying, which utilize both heat and moisture to promote the gelatinization process [17,18,19,20,21]. These methods influence sweet potato starch by altering its texture, viscosity, and functional properties. Different processing methods and conditions for the gelatinization of sweet potato starch are provided in Table 1, while Table 2 summarizes the characteristics of these various approaches. Understanding these techniques helps optimize the gelatinization process and provides insights into the application of gelatinized sweet potato starch in various foods. The following sections provide an overview of different moist thermal processing methods commonly employed for sweet potato gelatinization.

### 2.1. Moist Heat Processing

Moist heat processing is a widely used method where sweet potato starch is exposed to a large quantity of water and heat [34]. During this process, the starch granules absorb water, which initiates the swelling phase and is a crucial step in breaking down the native structure of the starch. This process facilitates rapid swelling and disruption of starch granules, resulting in gelatinization [7]. Sweet potato starch gelatinizes with an onset temperature of 59.72 °C, a peak of 71.85 °C, and a conclusion of 87.30 °C. These temperatures vary across starch types, influencing the properties of the gelatinized starch. Temperatures between 60 °C and 100 °C facilitate this process, with the optimal range being 90 °C to 95 °C to achieve efficient gelatinization while minimizing the risk of degradation. Extended heating beyond 30 min often results in adverse effects on the starch’s functional properties [35,36]. The interaction between starch and water is influenced by factors such as temperature, heating time, and the amount of water present [7]. Proper control of these factors is essential to ensure the desired texture and consistency of the gelatinized starch.

Boiling sweet potatoes may enhance gelatinization and contribute to a stronger gel structure; however, it significantly reduces their nutritional properties. Compared to sous vide cooking, boiling lowers the total phenolic content by 2.06 mg/g and decreases antioxidant activity, with DPPH and ABTS values dropping by 5.73 μM/g and 7.95 μM/g, respectively. These losses occur because heat-sensitive phenolic compounds are easily leached out during prolonged exposure to high temperatures and water immersion [4,14]. The improved gelation properties are beneficial in the production of chewy food products, where the ability to form strong gels is key to achieving the desired texture and mouthfeel. This enhancement contributes to its functional properties for use in various food applications [22,37]. Dietary fibers and phenolic compounds inherent in sweet potatoes also interact with starch granules under moist and high-temperature conditions. On one hand, these interactions may hinder localized swelling; on the other hand, they can provide diversified pathways at the granule–moisture interface. The physical effects and intermolecular interactions of these non-starch components offer new approaches for regulating starch structure, potentially enhancing overall gel performance while preserving the natural flavor and functional components of sweet potatoes.

### 2.2. Steaming

Steaming is another effective moist thermal processing method for sweet potato starch gelatinization [15,23]. Unlike boiling, where the starch granules are directly immersed in water, steaming involves subjecting the starch to indirect steam exposure, which minimizes direct contact with boiling water and thus reduces the leaching of soluble components [7]. During steaming, sweet potato cells generally maintain their structural integrity. However, cells with compromised walls gelatinize more easily, requiring less energy, as indicated by a gelatinization enthalpy of approximately 2.2 J/g lower than that of intact cells. This difference results in vigorous hydration on the granule surface while keeping the inner region compact and intact, preventing excessive decomposition or swelling during hydration [38]. Steaming sweet potato starch at around 100 °C for 20 to 30 min effectively gelatinizes the starch to a degree of 75.7%, which is slightly lower than the 83.8% achieved by boiling but higher than oven roasting (73.0%) and microwave roasting (67.7%) [39]. During steaming, the moisture content of the starch typically reaches about 60%, compared to 70% in boiled starch. This lower moisture content results in a moderate degree of gelatinization. Although steaming does not achieve as complete gelatinization as boiling, it offers the advantage of better preserving the starch’s nutritional and functional properties. Consequently, steaming is an effective method for promoting the gelatinization of sweet potato starch while maintaining its nutritional profile [40]. Additionally, the lower moisture content compared to boiling helps maintain the structural integrity of the starch granules, resulting in a moderate degree of gelatinization. While the gelatinization achieved by steaming may be less complete than that from boiling, it offers the advantage of better preserving the starch’s nutritional and functional properties [40]. The crystalline structure of sweet potato starch, predominantly classified as A-type crystalline, plays a significant role in its gelatinization behavior, influencing the specific gelatinization temperatures and enthalpy values associated with this starch [41]. These characteristics make steamed starch more suitable for applications where a moderate level of gelatinization is preferred.

In steamed cakes, sweet potato flour increases the storage modulus of the dough. When oat flour is added at a proportion of 15%, it raises the gelatinization enthalpy of sweet potato flour to 177.26 J/g. However, further increasing the oat flour content to 20% and 25% causes the gelatinization enthalpy to decrease [42]. Steaming combined with microwave heating enhances sweet potato starch gelatinization and significantly increases chlorogenic acid (124.53 mg/100 g), vanillic acid (13.96 mg/100 g), and antioxidant activity (by 25.17%) when applied at 500 W and 1700 W for 12 min [27]. The combination of these two methods provides a synergistic effect, where the rapid internal heating of microwave treatment complements the surface-level gelatinization achieved by steaming, resulting in an evenly cooked product with desirable textural properties. Moreover, steaming retains the granular structure and crystalline regions of sweet potato starch better than boiling, contributing to a firmer texture in the final product [43]. The reduced disintegration of starch granules during steaming also means that the final product retains more of its original flavor and nutritional components, providing a more wholesome eating experience.

### 2.3. Baking

During the baking process, the gelatinization of sweet potato starch is not solely aimed at obtaining gelatinized starch but occurs as a dynamic process accompanying the baking stages [25,44,45]. Heating is applied slowly and uniformly, allowing moisture to gradually penetrate the starch granules, transitioning ordered crystalline regions to amorphous areas, and subsequently forming a viscoelastic gel network [46,47]. With radio frequency combined with oven (RF-O) baking, the core temperature of sweet potatoes reaches 88 °C in just 15.1 min. This method also ensures improved temperature uniformity, effectively preventing starch degradation and promoting consistent gelatinization. RF-O baking significantly reduces the hardness of raw sweet potatoes from 1313.98 gf to 42.47 gf, while enhancing the a* and b* values by 7.54 and 11.85, respectively, due to the Maillard reaction. These improvements in texture and color highlight the efficiency of RF-O baking in facilitating starch gelatinization, preventing degradation, and ensuring high product quality [44].

Texture enhancement during baking is closely linked to the reorganization of starch chains under optimal baking conditions. At 180 °C with a baking time of 15 min, the heat promotes the natural rearrangement of starch chains, leading to moderate expansion of internal pores [48]. This structural adjustment contributes to better water retention, with crumb moisture content increasing to 31.12%, and improved elasticity, reflected in a specific volume of 2.19 cm^3^/g. These changes underscore the role of precise baking conditions in achieving desirable textural properties in baked goods [44,48]. Additionally, sugar serves not only as a flavor base but also indirectly regulates the structural formation trajectory by competitively binding water or affecting starch swelling behavior. When the sugar content is optimal, it not only maintains a good balance of internal moisture and sweetness but also subtly alters the hydrogen bond arrangement, making the starch network more stable and improving the overall texture and the sequence of flavor release [49].

### 2.4. Frying

During the frying process, sweet potato starch does not fully gelatinize but forms unique partially gelatinized regions under the combined effects of high temperature and moisture changes [50]. The application of pulsed electric field pretreatment at 1.2 kV/cm significantly influences the frying behavior of sweet potato chips [51]. Heating at 190 °C reduces oil content from 22–23% to 18% while increasing the browning index from 152.4 to 155.3. These improvements result from enhanced porosity and faster moisture evaporation, which promote a crisp surface and a soft interior [51]. It is commonly used in making fried foods such as sweet potato balls and French fries [52]. Moisture in the food and the high temperature of the oil together create a unique gelatinization process that influences the texture and mouthfeel of the final product [26,53]. Heat transfer becomes more efficient within starch granules, allowing deeper swelling and an expansion of partially gelatinized regions when the moisture content reaches 30%. Under these conditions, the pasting temperature drops from 64.9 °C to 57.7 °C, and peak viscosity decreases from 521 BU to 199 BU [54]. The elevated moisture also promotes amylose breakdown, increasing the proportion of shorter chains and further altering gelatinization and rheological properties [54]. This not only enhances the internal softness of the final product but also imparts a well-layered texture. Conversely, with excessively low moisture, rapid dehydration leads to quick surface crisping, while the interior remains hard due to inadequate swelling conditions. Excessive moisture, on the other hand, hinders oil penetration, concentrating heat energy in limited areas and resulting in over-charring of the surface and an interior [26,53]. Analyzing micro-region changes under different frying conditions assists in predicting the final texture during product design. By considering heat transfer and localized swelling mechanisms, rather than viewing frying solely as a high-temperature environment, a deeper understanding of the relationship between frying conditions, gelatinization distribution, and texture formation is achieved.

### 2.5. Microwave Processing

Microwave heating can rapidly elevate sweet potato starch to gelatinization temperature within an extremely short time, achieving fast drying by using internal moisture as the heat transfer medium [55]. Unlike traditional external heating methods, microwaves transmit energy through direct coupling with polar molecules via electromagnetic waves, significantly reducing cooking time and energy consumption while preserving the natural pigments and some heat-sensitive nutrients of sweet potatoes [56].

Microwave heating accelerates the gelatinization of sweet potato starch, improving solubility and viscosity while maintaining nutritional components. Using microwave vacuum drying, moisture content decreases from 81.12% to 2.7%, and porosity increases to 67.5%, significantly enhancing textural crispness and functionality [57]. Additionally, the ΔE value remains around 10, indicating minimal color changes compared to fresh samples, ensuring high-quality sensory attributes [57]. This retention of nutritional value makes microwave cooking an attractive option, especially for consumers seeking minimally processed, nutrient-dense foods. However, the distribution of heat and moisture during the microwave heating process is not entirely uniform, which can lead to temperature gradients within the product and result in some starch granules not fully gelatinizing, thereby affecting the final texture. This partial gelatinization causes microwave-dried sweet potato chips to exhibit slightly lower gelatinization levels compared to other methods [58,59]. Nevertheless, the rapid evaporation of moisture under microwave induction leads to the formation of a porous surface structure, which not only accelerates drying but also enhances the crispiness of the final texture. Sweet potato slices dried at 180 W microwave power combined with 70 °C hot air exhibited a reduced water-holding capacity of 28.44% and a rehydration ratio of 2.95, highlighting the structural changes responsible for the improved crispness [60]. Microwave-assisted processing often results in lower starch gelatinization compared to other methods due to uneven heat and moisture distribution, causing temperature gradients within the product. During microwave-assisted vacuum frying of purple-fleshed sweet potato slices at 1000 W and 90 °C for 15 min, the equilibrium moisture content decreased to 0.0452 at 60 °C from 0.0598 in untreated samples, while the monolayer moisture content dropped by 18.2%. Despite these challenges, the method significantly reduced processing time to 15 min and achieved better sensory qualities, including a crispier texture and reduced oil absorption, compared to freeze-dried products [27,61]. This is partly due to the unique way microwaves interact with the water molecules, leading to rapid moisture evaporation and the formation of an appealing, crispy texture. Moreover, the preservation of natural pigments during microwave processing results in a more native product.

### 2.6. Extrusion Processing

Extrusion is a high-temperature, short-time process where material is forced through a die under high pressure. The combined effects of heat, pressure, and mechanical shear result in structural and physicochemical changes in food materials [62,63]. This process is widely recognized for its efficiency in gelatinizing starch and its ability to produce modified textures suitable for diverse food applications [64]. The key mechanism driving gelatinization during extrusion involves the disruption of starch granules through heat and shear forces, leading to the loss of crystallinity and the formation of an amorphous structure. This process is heavily influenced by extrusion parameters, with specific mechanical energy reaching up to 157 kJ/kg at a screw speed of 140 rpm and a die diameter of 6 mm, while a lower specific mechanical energy of 69 kJ/kg was recorded at 80 rpm with a 10 mm die diameter. The extrudate temperature also varied between 97.25 °C and 122 °C, with higher screw speeds and smaller die diameters promoting more intense gelatinization due to increased energy input and shear forces [30,64]. Similarly, screw speed modulates shear intensity, which can improve gelatinization but may also reduce the molecular weight of starch polymers due to increased fragmentation. Optimizing these factors is crucial for achieving desirable product characteristics. Extrusion enhances gelatinization and helps form unique textures that are especially beneficial for snacks [65]. Moderate-temperature extrusion at 150–155 °C with a low moisture feed rate of 50.4–50.8 mL/min significantly improved the expansion ratio of sweet potato products to 2.35 mm/mm and specific volume to 4.84 cm^3^/g, enhancing their crispness and texture [29]. In contrast, processing at 180 °C strengthens the interaction between extruded purple sweet potato flour and gluten networks, yielding a higher specific volume of 2.44 mL/g and a lower hardness of 985.19 g, compared to the unextruded flour, which exhibits a specific volume of 2.17 mL/g and a hardness of 1636.50 g [66]. Mechanistically, the gelatinization of sweet potato starch during extrusion involves not only the hydration and swelling of starch granules but also complex molecular rearrangements. The applied shear stress aligns and fragments amylose and amylopectin chains, contributing to the distinctive textural properties of extruded products. Furthermore, extrusion facilitates the formation of a continuous starch matrix with enhanced structural stability, making it particularly advantageous for developing innovative products with customized sensory attributes.

### 2.7. High Hydrostatic Pressure

**Figure 1:**
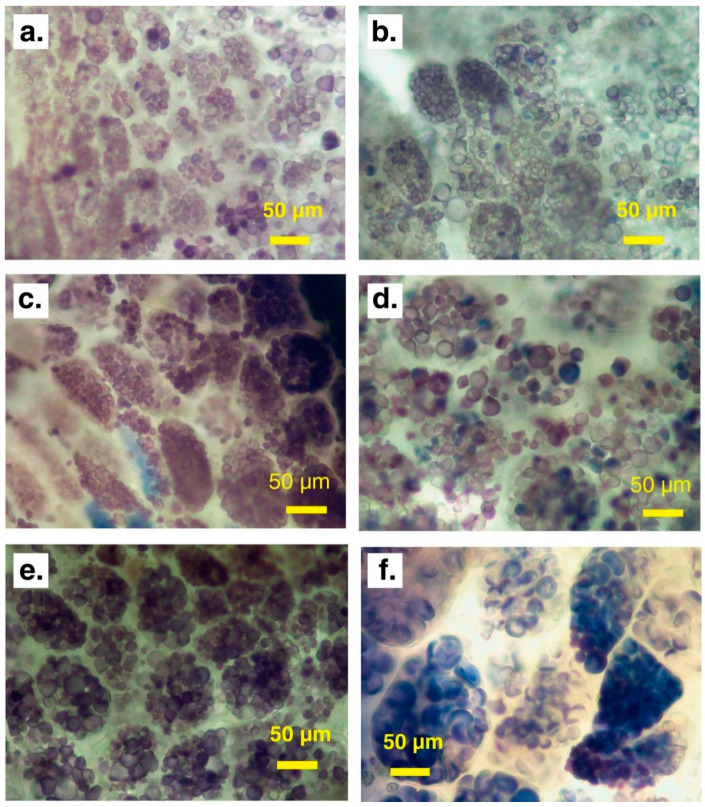
Microscopic structure of sweet potato starch granules treated with HHP for 10 min at various conditions: 200 MPa, 60 °C (**a**); 200 MPa, 70 °C (**b**); 500 MPa, 60 °C (**c**); and 500 MPa, 70 °C (**d**). Controls include untreated samples (**e**) and thermal treatment at 80 °C under atmospheric pressure (**f**). Starch stained with iodine solution. Bars = 50 μm [9] (Copyright 2017, Elsevier).

High Hydrostatic Pressure (HHP) is an innovative non-thermal technique that has been employed to gelatinize sweet potato starch. HHP uses very high pressures (typically 300–600 MPa) to induce structural changes without applying significant heat [67]. This helps the starch retain more of its preserving flavor, color, and bioactive compounds that are often degraded by thermal treatments [16,68,69]. Additionally, minimizing the loss of heat-sensitive nutrients makes HHP a preferred method for producing functional gelatinized starch. HHP is effective in enhancing the viscosity and gel-forming properties of sweet potato starch [31]. Controlled pressure treatment not only enhances the gelling ability of sweet potato starch but also significantly affects its rheological properties. At 600 MPa, the swelling of sweet potato starch granules is highly pronounced (Figure 1) [31]. In contrast, treatment at 500 MPa forms a well-structured network in the dough [16]. This network structure contributes to improved dough’s rheological properties [16]. Additionally, pH and inorganic salts profoundly influence the rearrangement of hydrogen bonds between starch chain segments, allowing for localized regulation of the gel’s elasticity and stability [70]. HHP at 600 MPa significantly accelerates drying and processing by disrupting cell walls and starch granules, enhancing moisture diffusion rates. In sweet potatoes, the drying rate increased by approximately 30%, and syneresis rose from 2.4% in untreated samples to 12.4% after treatment, reflecting notable structural changes [10]. Despite these modifications, flavor and functional component loss remained minimal, making HPP an effective non-thermal pretreatment method that balances nutritional and sensory qualities [10].

### 2.8. Spray Drying

Spray drying transforms sweet potato starch slurry into fine droplets, which are rapidly dehydrated and solidified in a high-temperature airflow to produce a powdered product [71]. During the spray drying process, sweet potato starch slurry is atomized into fine droplets that are typically exposed to temperatures above 100 °C [72]. This exposure enhances its solubility [32]. Spray drying of pregelatinized sweet potato starch preserves its gelatinization properties while enhancing usability and storage stability. Under optimized preheating conditions at 67 °C, the process achieves a high gelatinization degree of 71.75% and a process yield of 65%, ensuring uniform particle sizes with reduced moisture variability [73]. Starch granules transition from a dispersed state to highly aggregated microparticles, improving swelling power, which increases to 46.21 g/g compared to 37.04 g/g for native starch. These changes ensure better-thickening properties, viscosity, and stability for diverse industrial applications [73]. High temperatures promote partial or complete gelatinization of starch chains, accompanied by moisture evaporation that forms an interwoven network. When drying temperatures are high but residence times are short, moisture is rapidly removed in a high-energy environment, preserving a relatively intact microstructure, which helps maintain good solubility and rehydration performance [74,75]. An inlet temperature of 172 °C combined with a feed flow rate of 20 mL/min has been identified as optimal for achieving the highest yield during spray drying [75]. The sugars and other components in sweet potato starch interact during heat drying, influencing viscosity and rheological properties. Optimizing particle size distribution and internal pore structure allows for adjustments in parameters such as thickening rate and viscosity stability. With sucrose addition at a ratio of 3:1 (starch to sucrose), the pasting temperature increases from 75.6 °C to 76.6 °C, and at 3:3, it further rises to 77.4 °C. Breakdown viscosity also decreases from 99 mPa·s in native starch to 79 mPa·s, reflecting improved stability and enhanced functional properties for diverse applications [76,77]. Matching droplet morphology with the drying curve is critical in spray drying, as droplet size, viscosity, and the atomization method directly influence the dehydration rate and molecular interactions during high-temperature drying. Proper control of these parameters results in dried particles with improved uniformity and rehydration efficiency. Starch droplet sizes between 5 and 15 µm demonstrated higher rehydration rates, while particle density decreased to 0.42 g/cm^3^, enhancing functional characteristics. However, local overheating or aggregation during drying can disrupt this balance, leading to structural inconsistencies that negatively affect product quality [72,78].

## 3. Determinants of Gelatinization in Sweet Potato Starch

**Figure 2:**
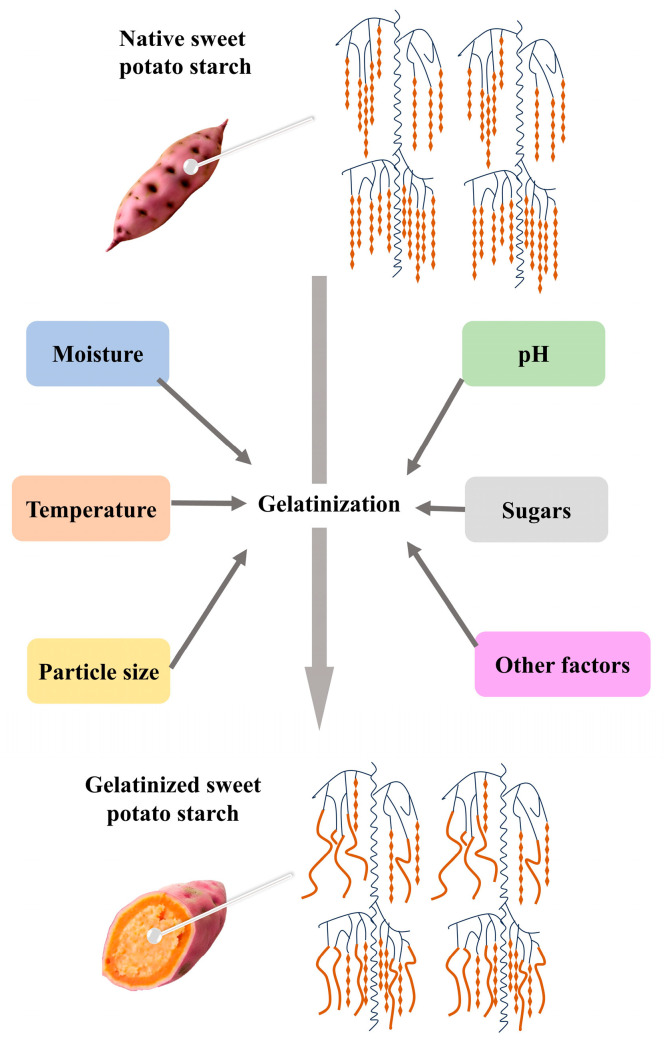
Factors affecting the gelatinization of sweet potato starch include moisture content, temperature, particle size, pH, and sugar.

The gelatinization process of sweet potato starch is influenced by various factors that determine its physical, chemical, and functional properties (Figure 2). Understanding these factors can help optimize the gelatinization process, enabling the application of gelatinized sweet potato starch in a wide range of food products (Table 3). The following sections explore the effects of factors such as the water-to-starch ratio, temperature, time, pH, and the presence of salts on the gelatinization of sweet potato starch.

### 3.1. Moisture Content

Moisture is one of the most crucial factors affecting the gelatinization of sweet potato starch [79]. Adequate water is essential for the proper hydration of starch granules, which swell and absorb water as they are heated, undergoing a transformation that leads to gelatinization. The degree of gelatinization is directly related to water availability [80]. Pre-cooking treatments that allow sweet potatoes to absorb water in advance can improve their gelatinization [79]. While it is well understood that adequate hydration promotes the orderly swelling and disruption of starch granules, leading to improved textures and product consistency, moisture control could be leveraged far more dynamically [13,92,93].

Moisture significantly influences the degree of gelatinization as well as the rheological, nutritional, and sensory properties of sweet potatoes. Steaming relies on the water within the sweet potato and the steam itself, resulting in a gelatinization degree of 41.4% in precooked and steamed samples, compared to 13% in those steamed directly. In contrast, boiling achieves the same degree of gelatinization but causes greater nutrient loss, with soluble cell wall components reaching 5.9% and uronic acids at 3.4%, compared to 3.9% and 1.2% in steamed samples. While steaming better preserves nutrients, it produces a less uniform texture and lower gelatinization efficiency [79]. In applications such as sweet potato starch pearls or gels, excessive moisture often results in undesirable characteristics such as overly soft textures, lack of elasticity, and poor chewiness, which diminish product quality and consumer satisfaction. Reducing water content during formulation or processing is essential to achieve the firm and elastic texture that defines high-quality starch pearls [94,95,96]. Beyond enabling granule swelling and structural disruption, precise moisture modulation could influence the interaction of starch with other constituents—such as proteins, lipids, and bioactive compounds—thereby altering nutrient bioavailability and the structural integrity of complex food matrices [63,96]. This approach encourages viewing moisture not merely as a backdrop to sweet potato starch gelatinization, but as a key driver of innovation. By strategically managing water content and distribution, it becomes possible to tailor the extent and nature of starch gelatinization, thereby shaping the textural, nutritional, and sensory attributes of the final product. In this way, moisture control in sweet potato starch systems can support product differentiation, unlock enhanced functionality, and inspire the exploration of novel applications previously overlooked in conventional starch science.

### 3.2. Temperature

Temperature is a key factor in determining the extent of sweet potato starch gelatinization. The typical gelatinization temperature of sweet potato starch ranges between 60–85 °C, with slight variations depending on the variety [35,97,98]. Higher temperatures provide the necessary energy to break down the crystalline structure of starch granules, thereby accelerating the gelatinization process [80]. However, temperature control is critical, as excessive heat can lead to starch degradation, negatively impacting its viscosity and gel strength [81]. This degradation is often accompanied by molecular fragmentation, reducing the average molecular weight and thus compromising the functional properties of the starch, such as its water-holding capacity and textural stability.

Treating sweet potatoes with superheated steam generates rapid heat, which can achieve 95% gelatinization at 140 °C, whereas baking requires a temperature of 240 °C to achieve the same effect [17]. Superheated steam treatment is advantageous because it minimizes nutrient loss, as the rapid heating reduces the time during which vitamins and other heat-sensitive compounds are exposed to high temperatures. Therefore, maintaining an appropriate temperature is essential to ensure efficient gelatinization while preserving the functional properties of sweet potato starch. In Japan, specific varieties of sweet potatoes with low gelatinization temperatures have been reported [99,100,101]. These varieties can gelatinize at lower temperatures, helping to retain more nutrients and natural flavors. Additionally, temperature uniformity during heating is crucial, as localized overheating may lead to uneven starch gelatinization, affecting overall product quality. When using microwave heating, not only should the peak temperature be controlled, but also the uniformity of the heating process to prevent unnecessary degradation [102]. A combination of appropriate temperature and time ensures that sweet potato starch reaches the desired level of gelatinization, resulting in optimal physical properties. Therefore, temperature control during heating is vital, as it impacts not only the degree of gelatinization but also the final product’s quality. Proper control prevents overprocessing, thereby maintaining the nutritional and sensory qualities of the sweet potato. In addition to temperature, other factors such as heating medium, humidity, and starch concentration also interact with temperature to influence gelatinization outcomes, highlighting the importance of a comprehensive approach to process optimization.

### 3.3. Amylose/Amylopectin Ratio

The ratio of amylose to amylopectin in sweet potato starch significantly influences its gelatinization properties. Higher amylose content raises the onset temperature of gelatinization from 73.5 °C in wild-type starch to 76.2 °C in waxy varieties, while the peak viscosity drops from 1235 cP to 694 cP. These changes are attributed to the rigidity of amylose molecules, limited granule swelling, and reduced water penetration, which weaken the post-gelatinization network. This results in lower transparency and decreased suitability for certain processing applications [103,104]. In contrast, sweet potatoes with a higher proportion of amylopectin, due to their branched structure, markedly enhance water binding ability, forming a more viscous and structurally stable paste. This characteristic lowers the gelatinization temperature while providing higher transparency and excellent freeze-thaw stability [105,106].

The amylose-to-amylopectin ratio and structure in sweet potato starch can be adjusted at the molecular level through genetic regulation and cultivation practices. Nitrogen application at 150 kg/ha led to a decrease in amylose content from 28.39% to 24.03%, accompanied by an increase in amylopectin content from 70.61% to 75.97%. This shift raised the amylopectin-to-amylose ratio from 2.49 to 3.16, improving starch functionality and broadening its potential for industrial applications [107]. High amylose content not only improves heat resistance but also affects the stability of the composite gel network [108]. Conversely, high amylopectin content is more suitable for thickening and emulsifying applications, as its branched structure rapidly forms viscoelastic networks with water molecules under heating and shear conditions [108]. Additionally, the distribution of long and short chains within amylopectin molecules is crucial; longer chains facilitate the formation of denser hydrogen bond networks during gelation, enhancing paste strength, while shorter chains reduce the energy required for gelatinization, making starch more easily swollen [109,110]. It is noteworthy that different sweet potato varieties inherently vary in the expression patterns and regulatory efficiency of starch synthesis genes, resulting in different starch component ratios and structural characteristics even under identical external conditions [111]. Therefore, a comprehensive consideration of a variety of traits, enzyme activity regulation pathways, and external cultivation conditions offers new possibilities for the targeted optimization of sweet potato starch gelatinization characteristics, thereby expanding its application potential in frozen, thickening, and various functional food developments.

### 3.4. Particle Size

The particle size of sweet potato starch plays a significant role in the gelatinization process. Because smaller particles have an increased surface area relative to volume, they facilitate more efficient water absorption and heat transfer which leads to faster and more effective gelatinization [112,113]. In contrast, larger particles may require more time and energy to fully gelatinize [112]. The particle size distribution of sweet potato starch is bimodal, with granules categorized as small (<2.27 μm), medium (2.27–17.51 μm), and large (>17.51 μm) [82]. Notably, a reduction in sweet potato particle size from 269 to 66 μm not only mitigates adverse effects on noodle quality but also enhances the orderliness of the secondary structure and the microstructure of the noodles [114]. This underscores the importance of optimizing particle size in sweet potato starch for improved food quality.

### 3.5. pH

The pH of the environment also affects the gelatinization of sweet potato starch. Acidic or alkaline conditions can alter the structure of starch granules, thereby influencing their water absorption and gelatinization capacity [83]. In an acidic environment, the hydrogen bonding interactions among starch molecules weaken, enhancing the swelling of starch granules and promoting water absorption. Under pH 4 conditions, the formation of double helical structures between gelatinized potato starch significantly enhances the gel properties [84]. Strongly acidic (pH 1.5) or strongly alkaline (pH 11.5) conditions can negatively impact gelatinization [115]. High concentrations of acid or base can cause hydrolysis of starch, leading to structural damage to the granules and reducing their water absorption capacity and viscosity [115]. This hydrolysis not only lowers the gelatinization temperature but may also result in a deterioration of the final product’s texture. For example, the swelling capacity of starch at pH 3.5 and 4.5 is significantly lower than that of native starch, thereby reducing its gel properties [116]. Controlling the pH value during the gelatinization of sweet potato starch is crucial to prevent excessive acidity from reducing the viscosity and texture of sweet potato products, ensuring precise control over the gelatinization properties of the starch.

### 3.6. Sugars

The presence of sugars significantly influences the gelatinization process of sweet potato starch [117]. Sugars compete with water molecules for binding, reducing the availability of water and thus lowering the hydration and swelling of the starch [85]. This competition raises both the gelatinization temperature and enthalpy, delaying the gelatinization process. Specifically, high concentrations of glucose, fructose, and sucrose raise the gelatinization temperature, thereby delaying the gelatinization process [118]. When sucrose, raffinose, and stachyose are incorporated, the pasting and gelatinization temperatures increase while viscosity decreases, giving the starch more fluid-like properties [77]. Research indicates that sucrose has the most pronounced effect in increasing the gelatinization temperature of sweet potato starch [77]. The interaction between sugars and starch molecules can also alter the starch’s structure, enhancing the stability of crystalline regions and thus improving the gel stability and texture of sweet potato products [77]. Additionally, the type and concentration of sugars can influence the stability and shelf life of the final product by reducing moisture migration and alleviating staling [119]. The addition of sugars has a notable impact on the gelatinization process of sweet potato starch, altering the interactions between starch and water through physical and chemical mechanisms, which, in turn, affects the gelatinization and retrogradation properties of the starch [77].

Several other factors influence the gelatinization of sweet potato starch, including the presence of salts, the varying amylose and amylopectin content among different sweet potato varieties, and the addition of lipids [80]. Salts can enhance gelatinization by stabilizing the starch granules and increasing the availability of water through ionic interactions, although their impact may vary based on concentration and type [88,89]. The composition of starch, particularly the ratios of amylose and amylopectin, also affects gelatinization, with higher amylose content generally leading to an increased gelatinization temperature [86,87]. Additionally, the presence of lipids can affect the hydration and swelling of starch granules, potentially inhibiting gelatinization if water availability is compromised [90,91]. While these factors are recognized for their influence on starch gelatinization, research specifically focused on their effects on sweet potato starch is limited. Further exploration of these factors could provide valuable insights for optimizing the gelatinization process and developing higher-quality sweet potato products.

## 4. Applications of Gelatinized Sweet Potato Starch in Food

Gelatinized sweet potato starch is primarily utilized as a functional additive, acting as a gelling agent and thickener in various food applications. Its ability to enhance texture and stability makes it particularly valuable in thermal processing [120,121]. It can be used in pasta, noodles, bread, and infant foods as a thickening, pasting, or gelling agent [122]. In the production of noodles, gelatinized sweet potato starch improves the cooking and textural properties, contributing to a firmer bite and better overall mouthfeel [123,124]. It also helps reduce the glycemic index of the noodles to 58.38 [123]. The gelatinization temperature of mixed flour increased from 66.31 °C to 67.73 °C with the addition of purple sweet potato flour, which further enhances noodle quality by improving paste viscosity, and stability, and reducing boiled loss, particularly at an optimal proportion of 15% purple sweet potato flour [125]. Moreover, heat moisture treatment of sweet potato starch enhances the quality of bihon-type noodles, with white sweet potato starch emerging as the best choice due to its low cooking losses and high color retention [126].

Transitioning beyond traditional uses, gelatinized sweet potato starch offers significant potential in other food categories, such as gluten-free bakery products. In these applications, it improves dough elasticity and cohesion, effectively mimicking the properties of gluten while enhancing moisture retention [16]. This makes it highly suitable for individuals with celiac disease or those seeking gluten-free options [16]. Additionally, in gluten-free bakery products, it not only improves dough elasticity and cohesion but also enhances the structural integrity of baked goods, offering a solution to the common challenge of brittle textures in gluten-free formulations [127]. By retaining moisture effectively, it extends the shelf life of products such as bread and pastries, which are often prone to drying out [128]. This makes it highly suitable for individuals with celiac disease or those seeking gluten-free options, while also aligning with growing consumer demands for functional and allergen-free foods. In dairy alternatives, such as plant-based yogurts, puddings, and frozen desserts, gelatinized sweet potato starch contributes to the desired creamy textures and ensures improved stability under a wide range of thermal and mechanical processing conditions [129,130]. Its ability to mimic the mouthfeel of dairy fats makes it a valuable ingredient for replicating the sensory qualities of traditional dairy products in plant-based formulations.

Beyond these applications, gelatinized sweet potato starch holds potential in innovative product categories. In plant-based meat analogs, it can improve water retention, texture, and mouthfeel, bridging the gap between plant-based and traditional meat products [131]. In sauces and gravies, it can provide viscosity and stability while resisting retrogradation, ensuring a consistent texture over time [132]. Moreover, its potential as a fat replacer in processed foods opens avenues for healthier formulations without compromising taste or texture [133]. The application of gelatinized sweet potato starch aligns with environmental and economic goals. Sweet potatoes are widely cultivated and relatively resource-efficient compared to other starch sources, making their use in food production more sustainable [134]. Furthermore, the potential for utilizing by-products, such as sweet potato peels rich in bioactives, enhances the environmental profile of sweet potato-based starch applications [135]. These by-products could be repurposed into functional food ingredients or natural additives, reducing waste and promoting circular food systems.

Gelatinized sweet potato starch is a vital functional additive with broad applications across various food categories, from noodles and baked goods to beverages and processed products. Its ability to enhance texture, stability, and nutritional value, combined with its role in promoting sustainable food systems, makes it a valuable ingredient in modern food innovation.

## 5. Conclusions and Future Perspective

The gelatinization of sweet potato starch enhances its structural and functional properties, expanding its applications in the food industry. Techniques such as moist heat processing, extrusion, and spray drying improve its crystalline structure, viscosity, and solubility, enhancing its performance in food formulations. Optimizing factors like moisture and temperature is crucial for producing high-quality starch, supporting its use in diverse products such as noodles and baked goods. Further exploration of innovative gelatinization methods and interactions with food components may improve nutritional profiles. Additionally, understanding its potential health benefits, including prebiotic effects and impacts on gut microbiota, will be vital for promoting its application in functional foods.

## Figures and Tables

**Table 1:** Effect of processing techniques on sweet potato starch.

Treatment	Parameters	Influence	References
Moist heat processing	60–80 °C	Cassava starch shows slight swelling and 8.5% amylose leaching, while potato starch swells rapidly with 51.05% amylose leaching	[22]
Steaming	140 °C	High gelatinization (up to 95%) without charring	[17]
Steaming	121 °C, 10 min	Maintained high antioxidant levels, preventing charring and increasing phenolic content	[23]
Baking	200 °C, 20 min	As maltose levels increase, starch levels decrease	[24]
Baking	200 °C, 40–50 min	Baking only slightly reduced the total anthocyanin content	[23]
Baking	200 °C, 90 min	Increased maltose content and sweetness, enhancing sensory acceptability	[25]
Frying	130 °C	Lower oil content by 15% compared to single-stage frying, improved texture and appearance	[26]
Microwave and Steaming	microwaving (1000 W) and steaming (1700 W)	Enhanced antioxidant activity, reduced cooking time	[27]
Explosion puffing drying	80 °C, 5 min	Enhanced anthocyanin retention and improved crispness, beneficial for gelatinization quality	[28]
Extrusion processing	150–155 °C	High sensory score and micronutrient retention, with optimal expansion	[29]
Extrusion processing	100 °C	Die diameter has a greater impact on product temperature than screw speed and feed composition	[30]
High hydrostatic pressure (HHP)	200–600 Mpa, 15min	Enhancing digestibility and bioactive compound extractability	[31]
Spray drying	Inlet temperature (130 °C), outlet temperature (105 °C)	Complete starch gelatinization is suitable for applications requiring rapid solubility and low final viscosity	[32]
Radio frequency blanching	90 °C	Improved enzyme inactivation, better color and texture retention	[33]

**Table 2:** Characteristics of starch gelatinization processing techniques.

Treatment	Mechanism	Advantages	Limitations	Applications	References
Moist heat processing	High temperature and abundant water cause starch granules to swell	Efficient gelatinization improves viscosity and gel-forming ability	High nutrient loss, high energy consumption	Sweet potato pearls	[22]
Steaming	Gradual moisture penetration, uniform gelatinization, retains more nutrients, lower degree of gelatinization than boiling	Better nutrient retention	Lower degree of gelatinization, higher equipment costs	Steamed pastries	[17,23]
Baking	Provides slow, uniform heating, suitable for baked products like bread and cakes, and improves texture and mouthfeel	Versatile for various baked goods	Dependent on other ingredients (additives and wheat flour), high energy consumption	Baked products like bread and cakes	[24,25]
Frying	Partial gelatinization through high oil temperature forms a crispy outer layer with a soft interior, suitable for foods like sweet potato balls and fries	Unique texture (crispy exterior, soft interior), fast processing	Partial gelatinization, increased fat content	Fried sweet potato balls, french fries	[26]
Microwave heating	Rapid energy transfer for quick gelatinization retains more nutrients but may face uneven heating challenges	High energy efficiency, good nutrient retention	Uneven heating, industrial scaling challenges	Rapid dehydration and texture improvement products	[27]
Extrusion processing	High pressure and temperature with mechanical shear induce gelatinization	Efficient gelatinization	High equipment costs, complex operation	Puffed sweet potato products	[29]
High hydrostatic pressure	Low-temperature treatment prevents nutrient degradation, enhances starch viscosity and gel properties, retains natural flavor and color	Preserves nutrients and sensory qualities	Longer overall processing time	Products requiring retained nutrition and sensory attributes	[31]
Spray drying	Atomizes starch slurry into droplets for quick drying into powder, improves solubility and viscosity, convenient for storage and application	Convenient powder form, enhanced solubility, and rheological properties	High energy consumption	Instant food ingredients, thickening agents, or stabilizers in various food applications	[32]

**Table 3:** Key factors influencing starch gelatinization.

Influencing Factors	Mechanism	References
Moisture content	Adequate water facilitates the hydration and swelling of starch granules, disrupting crystalline structure. Insufficient moisture leads to incomplete gelatinization	[79,80]
Temperature	Higher temperatures break crystalline structures, accelerating gelatinization. Excessive heat can degrade starch, reducing viscosity and gel strength	[80,81]
Particle Size	Smaller particles increase surface area, allowing faster water absorption and heat transfer, leading to efficient gelatinization	[82]
pH	Acidic or alkaline conditions alter hydrogen bonding and starch structure, affecting water absorption and gelatinization. Extreme pH can cause hydrolysis and reduce functional properties	[83,84]
Sugars	Compete with starch for water, reducing swelling and delaying gelatinization. Increase gelatinization temperature and improve stability in final products	[77,85]
Amylose/Amylopectin ratio	Higher amylose content increases gelatinization temperature and affects texture. Amylopectin-rich starches gelatinize more easily	[86,87]
Salts	Stabilize starch granules and enhance water availability through ionic interactions, influencing gelatinization degree	[88,89]
Lipids	Interact with starch to form complexes, reducing water availability and potentially inhibiting gelatinization	[90,91]

## Data Availability

No new data were created or analyzed in this study. Data sharing is not applicable to this article.
